# The Role of Capsaicin-induced Acute Inactivation of C-fibers on Tactile Learning in Rat

**Published:** 2013-02

**Authors:** Mohammadreza Rahmani, Soodeh Rajabi, Mohammad Allahtavakoli, Ali Roohbakhsh, Vahid Sheibani, Ali Shamsizadeh

**Affiliations:** 1 Physiology-Pharmacology Research Centre, Rafsanjan University of Medical Sciences, Rafsanjan, Iran; 2 Neuroscience Research Center, Kerman University of Medical Sciences, Kerman, Iran; 3 Pharmacy Research Centre, School of Pharmacy, Mashhad University of Medical Sciences, Mashhad, Iran

**Keywords:** C-Fibers, Capsaicin, Learning, Recognition, Tactile

## Abstract

***Objective(s):*** In our previous study, we reported that capsaicin-induced unmyelinated C-fiber depletion can modulate excitatory and integrative circuits in the somatosensory cortex following experience-dependent plasticity. In this study, we investigated the involvement of the capsaicin-induced acute inactivation of c-fibers on tactile learning in rat.

***Materials and Methods: ***The delayed novel object recognition test was used to assess tactile learning. This procedure consisted of two phases. The first of these (T1) was a training phase during which the animals explored two similar objects. T2, the test phase, occurred 24 hr later, during which the animals explored one novel and one familiar object. In order to induce acute inactivation of the C-fiber pathway, 25–30 μl of a 10% capsaicin was injected subcutaneously into the rat’s upper lip, 6 h prior to T1. Tactile learning was quantified using a discrimination ratio.

***Results:*** In T2, the discrimination ratio in capsaicin-treated animals (37.3±3.8%) was lower than that observed in vehicle-treated animals (54.4±5.1%, *P*<0.05).

***Conclusion:*** These findings indicate that the selective inactivation of a peripheral nociceptor subpopulation affects tactile learning.

## Introduction

One of the key attributes of the cortex is plasticity, a phenomenon that allows us to adapt our behavior in the light of experience, including the formation of new memories (1).

The somatosensory cortex is the key to the integration and analysis of sensory information, leading to the perception of somatosensory stimuli. Through interactions with other areas in the brain such as the striatum and motor cortex, the somatosensory cortex enables planning, execution, and dynamic modulation of coordinated movement (2, 3). 

There is substantial evidence that unmyelinated fiber nociceptors play an important role in modulating receptive field properties of somatosensory neurons (4, 5). 

Previous studies in rats have shown that administration of capsaicin, the active ingredient of the pungent *capsicum* pepper, causes temporal inactivation (6) or permanent degeneration (7) of a significant fraction of unmyelinated primary sensory neurons, with no significant proportion in myelinated afferent fibers (8). Capsaicin induces receptive field changes when it is applied directly to the peripheral nerve (9, 10), injected subcutaneously (11, 12), or injected systemically into neonates (13, 14). Receptive field changes induced by these means have been detected at different levels of the central nervous system, including the cortex (12, 15), brain stem (14, 16), and spinal cord (11, 17).

In a previous study, we demonstrated that following the induction of experience-dependent plasticity, changes in excitatory and integrative circuits in the somatosensory cortex could be further influenced by capsaicin treatment (18). Here, we investigated the effect of acute inactivation of the nociceptive pathway on tactile learning, by means of a delayed novel object recognition test. Our results revealed that injection of small amounts of capsaicin 6 h before the commencement of training impaired recognition memory.

## Materials and Methods

*Animals*

A total of 30 male Wistar rats, weighing 170-210 g, were used for these experiments. The animals were allowed food and water ad *libitum*, and were housed in standard cages with a 12 hr light-dark cycle (lights on: 0700-1900 hr), with the laboratory temperature set at 23±2.0°C. The experimental protocols used in this study were approved by the Ethics and Animal Care Committee of Rafsanjan University of Medical Sciences and were performed in accordance with the National Institutes of Health Guidelines for the Care and Use of Laboratory Animals. 

*Object recognition task*

The test apparatus was a Plexiglas arena (35 ×35 ×35 cm) with a black plastic floor, placed in a dimly illuminated room (19, 20). The objects to be discriminated were square and triangular blocks made of iron. The rats’ behaviour was recorded by a camera positioned directly above the arena and subsequently analysed using Ethovison Software (Noldus, Wageningen, Netherlands). The object recognition task was done in three phases. On the first day, rats habituated to the empty apparatus for 30 min. Twenty-four hr later, the training (T1) phase was initiated. Each rat was placed in the arena with two identical objects, and allowed to explore for 5 min. The position and shape of the objects were changed between animals to prevent an order or side preference affecting the results. All rats were introduced into the arena at the same point, and facing the same direction. The test phase (T2) was conducted 24 hr after T1. During T2, each rat was returned to the arena which contained the familiar object, the position of which was consistent in both trials, and a novel object, for 5 min. To avoid the presence of olfactory cues, the box and objects were thoroughly cleaned with 70% ethanol between rats (21, 22). The time spent exploring each object and the total time spent exploring both objects were recorded. Exploration of an object was defined as pointing the nose to the object at a distance≤2 cm. Climbing or sitting on an object was not considered exploration. A discrimination index was calculated based on the difference in time exploring the novel and familiar objects, expressed as the ratio of the total time spent exploring both objects. 

*Capsaicin treatment*

The rats were injected with either capsaicin solution (25-30 µl of 10% capsaicin dissolved in 10% Tween 80, 10% ethyl alcohol, and 80% saline) or vehicle. Injections were made subcutaneously through a 30-gauge needle into the upper lip, 6 mm away from the whisker pad, nearest to whiskers E2 and E3 (6). This occurred 6 hr before the commencement of the T1 phase of the novel object recognition test and under light ether-induced anaesthesia.

*Experimental groups*

The rats were randomly allocated into the following experimental groups (10 rats per group). Group 1 animals received capsaicin 6 hr before the commencement of T1. Group 2 animals received vehicle 6 hr before the commencement of T1. Group 3 was a control group in which no injection was performed. 

*Statistical analysis*

 Data were analyzed for statistical significance using ANOVA. Data are expressed as mean±SEM. A *P-*value<0.05 was taken to be significant. All post-hoc comparisons were made using Tukey’s post-hoc test. Paired-sample t-test was used for comparing travelled distance between T1 and T2.

## Results

Activity levels were assessed by measuring the distance travelled during training phase (T1) and test phase (T2). 

**Figure 1:**
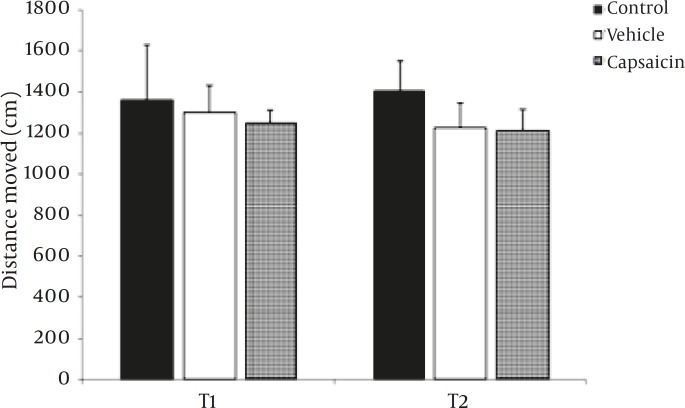
Comparison of the activity level in three experimental groups. Activity levels measured as distance travelled in 5 min during the T1 and T2 phases (21).

Comparing the travelled distance in T1 and T2 for all three groups represents no significant differences between two phases (in vehicle group *P=*0.2, in capsaicin-treated group *P=*0.7, and in control group *P=*0.8) (Figure 1). In the capsaicin treated animals, the travelled distance was not different compared with vehicle-treated group (in T1, *P=*0.8 and in T2, *P=*0.5) (Figure 1). 

*Object recognition task: training phase (T1)*

**Figure 2:**
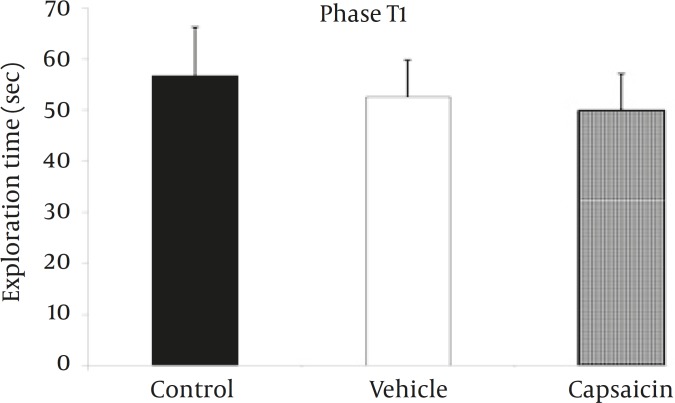
Time spent exploring objects in the phase T1 of the object recognition task.

**Figure 3:**
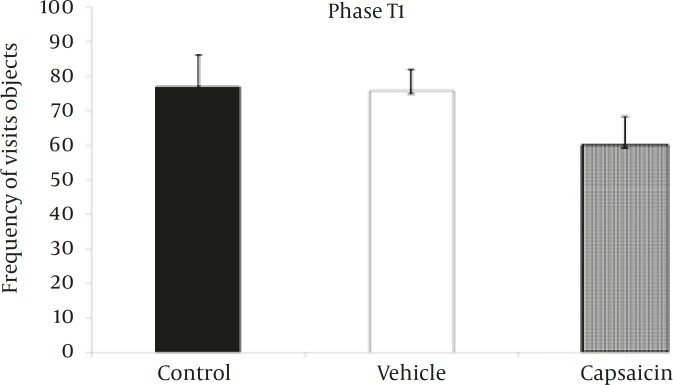
Frequency of visits to the objects in the phase T1 of the object recognition task.

The total time spent exploring sample objects in T1 (Figure 2) was not statistically significant between three experimental groups (*P=*0.8). During T1, no reliable differences were found among the three experimental groups (Figure 3) for the frequency of visits to the sample objects (*P=*0.2).

All data are expressed as mean±SEM

T1: training phase, T2: test phase

All data are expressed as mean±SEM

T1: training phase, T2: test phase

All data are expressed as mean±SEM

T1: training phase, T2: test phase

*Object recognition task: test phase (T2)*

**Figure 4:**
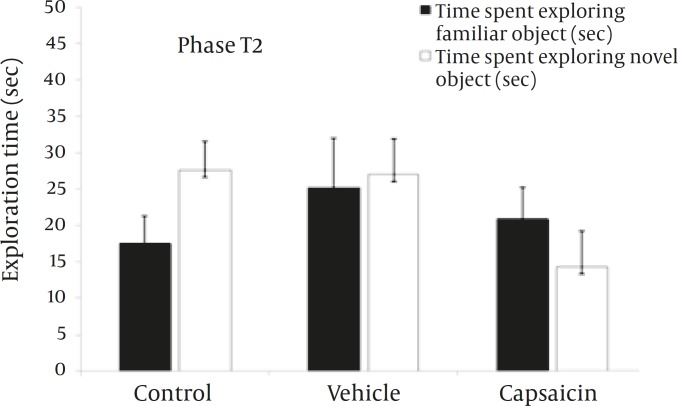
Time spent exploring the familiar and novel objects in the phase 2 of the object recognition task.

Object exploration times for the experimental groups during the test phase (T2) are shown in the Figure 4. The means (mean±SEM) of total exploration time of both objects (familiar+novel) were 45.1±6.9 sec (control group), 52.9±9.7 sec (vehicle-treated group), and 35.3±8.8 sec (capsaicin-treated group). Differences among these groups were not significant (*P=*0.4).

All data are expressed as mean±SEM

T1: training phase, T2: test phase

In the capsaicin-treated group, the mean time spent exploring the novel object (14.3±4.9 sec) was less than that observed for the control (27.6±4 sec) and vehicle-treated (27±4.9 sec) groups although the difference was not statistically significant (*P=*0.1).

**Figure 5:**
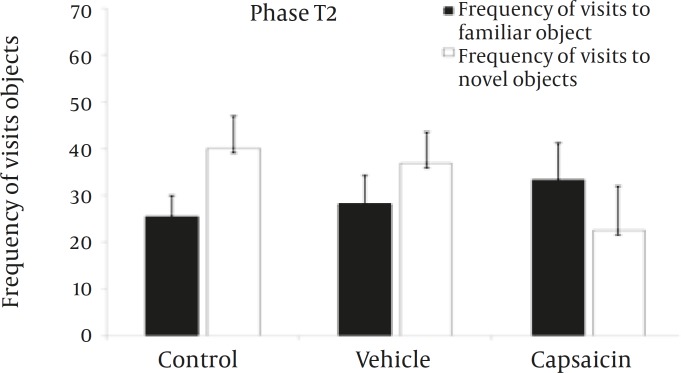
Frequency of visits to familiar and novel objects in the phase 2 of the object recognition task.

All data are expressed as mean±SEM

T1: training phase, T2: test phase

During T2, no reliable differences were found between the three experimental groups for the frequency of visits to the sample objects (Figure 5).

**Figure 6:**
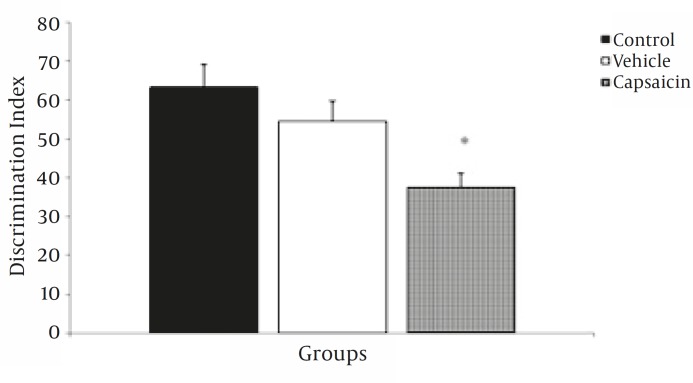
Effect of capsaicin or vehicle administration 6 hr before T1 on discrimination index.

Comparison of the discrimination ratio among the three experimental groups revealed that this index was lower for capsaicin-treated animals (37.3±3.8%) than for vehicle-treated (54.4±5.1%) animals (*P<*0.05) (Figure 6). These findings indicate that the ability to discriminate between familiar and novel objects is impaired in capsaicin-treated animals.

* A significant difference in the discrimination index between capsaicin-treated animals and vehicle-treated group (P<0.05).

Data are expressed as mean±SEM

## Discussion

The results of this study indicate that capsaicin induced acute inactivation of C-fibers impaired tactile learning in rats.

The receptive field properties of low threshold somatosensory barrel cortex cells are modulated by inputs conveyed by unmyelinated C-fibers (23, 24). In our previous study, we reported that following induction of experience-dependent plasticity in barrel cortex, these fibers can modulate excitatory and integrative circuits in the barrel cortex cells (18). In this study, we demonstrated that their acute inactivation can also change behaviors that are mediated by the somatosensory system, such as tactile learning.

Katz *et al.* (1999) reported that injecting capsaicin under the skin of the lip triggered increases in spontaneous firing rate and changes in the receptive fields of thalamocortical neurons, as well as barrel cortex cells. These capsaicin-related changes continued to emerge for more than 6 hr after the injection, and reorganization in the receptive fields of cells in both the cortex and thalamus was associated with a lessening of the “spatial coupling” between cortical neurons (6). In our previous study, we also demonstrated that neonatal capsaicin-induced C-fiber depletion can modulate the changes in excitatory and inhibitory receptive field properties observed as a result of experience-dependent plasticity (18). The findings of the present study are in good agreement with these electrophysiological data, revealing the behavioral consequences of selective inactivation of a peripheral nociceptor subpopulation.

In humans, application of capsaicin cream to the skin of the hand has been shown to affect two-point discrimination, and impair the ability to detect differences in roughness (25). Carrillo *et al* (1994) have also reported that neonatal capsaicin treatment causes a significant increase in scratching, rearing, grooming, and searching behaviors in rats. Furthermore, in a recent study, Fan *et al.* (2009) (26) demonstrated that applying capsaicin to the sciatic nerve differentially blocks nocifensive components of behavior such as flinch, withdrawal, and licking, but not non-nocifensive responses including slow body motion, turning, running, or exploration involving translocation of the body. However, as these authors recorded the rats’ behavior for only 2 min, and used body translocation for assessing exploratory behaviors, further studies are needed to clarify the behavioral significance of the nociceptor pathway.

*Study limitation*

There are some reports that capsaicin induces behavioral responses like itching and licking associated with pain in the region (27-29). Therefore, the capsaicin could have a direct effect on the whiskers tactile region altering the sensitivity of the rats to nociceptive and mechanical stimulation. However, other studies reported that applying capsaicin to the sciatic nerve differentially blocks nocifensive components of behavior such as flinch, withdrawal, and licking (26). Further studies are needed to address this issue. When we injected capsaicin subcutaneously into the rat’s upper lip, 6 hr prior to T1, some of them remained less active even 6 hr after we recorded training phase. In order to conclude that the capsaicin interferes with tactile learning, it is required that activity level and the total exploration time during the T1 be similar for all groups. Therefore, we discarded these animals from our study. 

## Conclusion

In summary, when taken together, the results of this and previous studies (6, 18, 23, 24) demonstrate that nociceptor information may be important in modulating both behavioral aspects of somatosensory function and electrophysiological properties of cells in somatosensory cortex. 
